# Ancient Chinese Character Recognition with Improved Swin-Transformer and Flexible Data Enhancement Strategies

**DOI:** 10.3390/s24072182

**Published:** 2024-03-28

**Authors:** Yi Zheng, Yi Chen, Xianbo Wang, Donglian Qi, Yunfeng Yan

**Affiliations:** 1 College of Electrical Engineering, Zhejiang University, Hangzhou 310027, China; zhengyipds@zju.edu.cn (Y.Z.); morningone@126.com (Y.C.); 2 Hainan Institute of Zhejiang University, Sanya 572025, China; xianbowang@zju.edu.cn; 3 School of Mechanical Engineering, Zhejiang University, Hangzhou 310027, China

**Keywords:** ancient Chinese characters, Swin-Transformer, cultural heritage, character recognition

## Abstract

The decipherment of ancient Chinese scripts, such as oracle bone and bronze inscriptions, holds immense significance for understanding ancient Chinese history, culture, and civilization. Despite substantial progress in recognizing oracle bone script, research on the overall recognition of ancient Chinese characters remains somewhat lacking. To tackle this issue, we pioneered the construction of a large-scale image dataset comprising 9233 distinct ancient Chinese characters sourced from images obtained through archaeological excavations. We propose the first model for recognizing the common ancient Chinese characters. This model consists of four stages with Linear Embedding and Swin-Transformer blocks, each supplemented by a CoT Block to enhance local feature extraction. We also advocate for an enhancement strategy, which involves two steps: firstly, conducting adaptive data enhancement on the original data, and secondly, randomly resampling the data. The experimental results, with a top-one accuracy of 87.25% and a top-five accuracy of 95.81%, demonstrate that our proposed method achieves remarkable performance. Furthermore, through the visualizing of model attention, it can be observed that the proposed model, trained on a large number of images, is able to capture the morphological characteristics of ancient Chinese characters to a certain extent.

## 1. Introduction

Chinese characters, as the sole extant logographic script still in active use, boast a venerable history spanning 3000 years [1,2]. Represented by oracle bone script and bronze inscriptions, the past century has witnessed a continuous influx of unearthed archival materials inscribed in ancient Chinese scripts. These materials provide a comprehensive panorama of ancient society, meticulously recording a diverse spectrum of societal activities, including religious practices, astronomical observations, calendrical systems, legal codes, and commercial transactions. Consequently, the scholarly exploration of ancient Chinese scripts holds not only paramount significance within the realm of Chinese philology but also constitutes a profound contribution to the inquiry into Chinese history and culture, and, by extension, the broader tapestry of human history.

The existing Chinese character recognition methods are mainly based on optical character recognition technology [3,4,5]. However, the common problem faced by this type of method is that these symbols differ greatly from modern Chinese. The evolution of Chinese characters can be divided into two phases: the Ancient Script Phase and the Clerical and Regular Script Phase [6]. The Ancient Script Phase began during the Shang Dynasty (11th century BCE) and persisted until the Qin Dynasty (late 3rd century BCE), while the Clerical and Regular Script Phase commenced during the Han Dynasty (2nd century BCE) and has continued to the present day. The characters from the Ancient Script Phase, which are precisely the Chinese ancient characters referred to in this article, have a more distant historical origin, exhibiting a stronger ideographic nature. In outward appearance, modern characters differ substantially from those of the ancient Chinese scripts. Meanwhile, due to the wide span of historical periods, ancient Chinese characters also exhibit variations in their writing styles across different periods. The ancient forms of commonly used characters from different periods are shown in Table 1. These characters represent “马mǎ” (horse), “牛niú” (cow/ox), “明míng” (bright), and “走zǒu” (run), respectively. Geographically, characters from different regions also feature distinctive characteristics. The different writing styles of the characters “者zhě” (someone who) and “市shì” (market) in various regions are shown in Table 2. Only linguists with prolonged and specialized training can recognize these ancient characters. This situation poses a significant hindrance for the general public and enthusiasts of ancient scripts in understanding and learning the historical evolution of each Chinese character.

Although some oracle bone script datasets have been made public [7] and significant progress has been achieved in oracle bone script recognition using computer vision techniques [5,8], for other styles of ancient characters, such as bronze inscriptions, coin inscriptions, stone inscriptions, and bamboo slip, wooden tablet, and silk manuscript texts, there is still no comprehensive, large-scale image dataset for common ancient Chinese characters. The advantage of building such a dataset is that it can help the public read various types of ancient texts. In addition, from a linguistic perspective, ancient Chinese from different eras can be connected. Although their appearance may differ, they have inherent commonalities in their character structure, which is also worth further research.

For the above purposes, we construct a large-scale dataset of ancient Chinese script images, totaling over 970,000 images, covering various types of ancient scripts such as oracle bone inscriptions, bronze inscriptions, large seal scripts, small seal scripts, and bamboo and silk texts. In the processing phase of the aforementioned database, we propose a model for ancient character recognition based on the Swin-Transformer. This recognition model utilizes the Swin-Transformer for feature enhancement and introduces a Context-Transformer (CoT) to enhance the learning ability of the enhanced features. The proposed model can reduce the resolution of the input feature map and gradually expand the perceptual field layer by layer. In summary, this work makes the following contributions:

- We collect various forms of each ancient script to comprehensively showcase the diversity of ancient Chinese characters and construct the first large-scale Chinese ancient script dataset, comprising 9233 classes and over 970,000 instances.
- This paper proposes an improved Swin-Transformer model for feature extraction and adopts a data augmentation strategy to solve the problem of long tail distribution in ancient Chinese characters.
- According to the latest research findings, this work is the first to apply deep learning models to recognize ancient Chinese characters on a large dataset. The primary goal of this work is to develop a deep learning network to analyze the inherent commonalities of ancient Chinese characters.

The rest of this paper is organized as follows: Section 2 analyzes the existing research on incident Chinese script recognition and image processing models. Section 3 explains the proposed architecture and implementation method based on the Swin-Transformer and improves the incident Chinese data enhancement strategy. Section 4 validates the proposed algorithm and compares the experimental results. Finally, concluding remarks are provided in Section 5.

## 2. Related Works

### 2.1. Ancient Chinese Script Recognition

In the realm of research on ancient Chinese script recognition methods, there has been notable scholarly attention devoted to oracle bone script recognition. Early oracle bone script recognition methods primarily involved the analysis of the topological structure of oracle bone script and employed techniques like support vector machines (SVMs) and fractal geometry for classification [9,10]. Regarding bronze inscriptions, Zhao et al. [11] developed an image recognition algorithm utilizing a histogram of oriented gradient (HOG) and a gray level co-occurrence matrix (GLCM) to address their morphology. Bilateral filtering was applied for preprocessing and extracting HOG and GLCM-based features, which then were fused into a combined feature set. This set was used to train an SVM classifier for recognizing bronze inscriptions. This method faces challenges related to limited model generalization ability.

In the past decade, deep learning technologies have made significant progress in the field of computer vision. An increasing number of researchers are exploring the use of deep learning methods for ancient script recognition. Some models that have shown significant effectiveness in the image domain, such as AlexNet, VGG, ResNet, and Inception-v3 [3,4,12,13], have been applied to the task of ancient script recognition, yielding impressive results. Guo et al. [14] combined features extracted by the convolutional neural network (CNN) with their proposed representation to further improve recognition accuracy. Lin et al. [15] improved the recognition accuracy of Oracle bone script using data augmentation methods. Liu et al. [16] described a method based on an adapted CNN, which can obtain the predicted recognition performance with a top-five accuracy of 94.38. Wang et al. [5] proposed a structure-texture separation network (STSN), which is an end-to-end learning framework designed for joint disentanglement, transformation, adaptation, and recognition. It successfully performs domain adaptation to transfer knowledge from handprinted oracle data, and it has also demonstrated promising results in recognizing Handprint oracle scripts and Scan oracle scripts. Li et al. [8] introduced a generative adversarial framework to improve the performance on the long-tailed oracle dataset and obtained remarkable performance in oracle character recognition. Wu et al. [17] explored modified CNN models to achieve bronze inscription characters. He et al. [18] employed a CNN to design a classification algorithm based on ResNet and mitigated the issue of imbalanced dataset distribution using the Two-Phases-Training method. In addressing the imbalance of training data in bronze inscription recognition, Zheng [19] proposed a recognition method with distribution calibration based on few-shot learning.

### 2.2. Image Classification Based on Convolutional Neural Networks

Since the proposal of AlexNet [20], CNNs have rapidly become a very popular technology in the field of computer vision. They possess representation learning capabilities, generalization capabilities, and translational invariance, efficiently handling large-scale images and transforming structural data. This has led to a qualitative development of CNNs, resulting in significant achievements in tasks such as image classification, object recognition, semantic segmentation, and more. Classical CNN models include VGGNet [21], GoogLeNet [22], ResNet [23], and DenseNet [24], among others. VGGNet improves performance by increasing the network’s structure, exhibiting good transfer learning capabilities. It utilizes small convolutional kernels instead of larger ones, reducing parameters and enhancing feature learning abilities. GoogLeNet incorporates the Inception structure, which is capable of fusing feature information at different scales, effectively improving network performance and efficiency. Subsequently, the Inception structure has been continuously improved [25,26,27], eventually leading to the Xception [28], optimizing the performance of the recognition model. The ResNet model extends the network structure to 1000 layers and improves the significant performance. Its residual modules effectively address the vanishing gradient problem and reduce parameters. The use of Batch Normalization also accelerates the convergence. DenseNet is composed of dense blocks, where each layer is directly connected in a feedforward manner. Each layer receives inputs from all preceding layers and passes its output feature map to subsequent layers. This approach enables the repeated use of features, addressing the issue of vanishing gradients.

### 2.3. Vision Transformer

In recent years, Vision Transformers (ViTs) [29] have demonstrated comparable performance to CNNs based on the Transformer architecture. The ViT stands out as the first model entirely built on the Transformer architecture, achieving state-of-the-art performance in the visual domain on large datasets such as ImageNet-22K and JFT-300M. Thanks to the success of ViTs, more models based on Transformer architecture have been designed for various downstream tasks. In recent developments in visual Transformers, the detection Transformer [30] emerged as a remarkable design based on the Transformer architecture, creating the first end-to-end object detection model. The data-efficient image Transformer [31] introduces efficient data training strategies and knowledge distillation, enabling ViTs to perform well even on smaller datasets (e.g., ImageNet-1K).

However, the ViT still involves significant training expenses. It outputs only a low-resolution feature map, which does not match the resolution of the prediction target. Some approaches have adapted the ViT architecture to support a wide variety of downstream visual tasks, such as semantic segmentation and object detection. The Segmentation Transformer [32] treats the Transformer as an encoder, simulating global context at each layer and combining it with a simple decoder to form a semantic segmentation network. The Pyramid Vision Transformer (PVT) [33] introduces a pyramid structure into the ViT to obtain multi-scale feature maps, mimicking the characteristics of CNN backbones. Specifically, it flexibly controls the length of the Transformer sequence using patch embedding layers. Although PVTs reduce the consumption of computational resources to some extent, their complexity still scales quadratically with image size. The Swin-Transformer [34], on the other hand, achieves linear computational complexity by introducing Window-based Multi-head self-attention (W-MSA) and Shifted Window-based Multi-head self-attention (SW-MSA). This innovation has led to state-of-the-art performance in image recognition and dense prediction tasks like object detection and semantic segmentation.

Unlike most previous Transformer-based models, the Swin-Transformer employs a hierarchical architecture for dense prediction, serving flexibly as a universal backbone network. The subsequent Swin-Transformer model introduces hierarchical structures and sliding window mechanisms, effectively addressing the challenges of excessive parameters and training difficulties faced by vision Transformers. By partitioning images into equally sized windows and performing information interaction only within the windows, it significantly reduces computational complexity, enabling linear scalability with image resolution. For tasks such as image classification, object detection, and semantic segmentation, visual Transformer models have successfully surpassed convolutional frameworks for the first time.

## 3. Methodology

### 3.1. Preliminaries

#### 3.1.1. Swin-Transformer

The Swin-Transformer extends the ViT model by incorporating a sliding window mechanism to facilitate the learning of cross-window information. Simultaneously, it employs a down-sampling layer to enable efficient processing of super-resolution images, conserving computational resources and focusing on both global and local information. The initial step involves dividing an input RGB image into non-overlapping patches, akin to ViTs, using a patch-splitting module. Each patch is treated as a ‘token’ with its feature representing the concatenation of raw pixel RGB values. A linear embedding layer is then employed to transform this raw-valued feature into an arbitrary dimension. The subsequent application of several Transformer blocks, which include featuring modified self-attention computation (e.g., Swin-Transformer blocks), is carried out on these patch tokens. These Transformer blocks maintain the number of tokens and work in conjunction with the linear embedding layer.

#### 3.1.2. Swin-Transformer Block

The Swin-Transformer is constructed by substituting the conventional MSA module in a Transformer block with a module grounded in shifted windows while leaving other layers unchanged. Specifically, a Swin-Transformer block comprises a shifted window-based MSA module, succeeded by a 2-layer MLP with GELU non-linearity interspersed between the layers. A LayerNorm (LN) layer precedes each MSA module and each multilayer perceptron (MLP), and a residual connection is applied after each module.

#### 3.1.3. Shifted Window-Based Self-Attention

Initially, the self-attention within local windows is computed, with the windows arranged to evenly partition the image in a non-overlapping manner. Assuming each window contains *M* × *M* patches, the computational complexity of a global MSA module and a window-based MSA module on an image of *h* × *w* patches is

(1) $$\Omega \left(\mathrm{MSA}\right)=4hwC^{2}+2{\left(hw\right)}^{2}C$$

(2) $$\Omega \left(W\text{-}\mathrm{MSA}\right)=4hwC^{2}+2M^{2}hwC$$

where the former is quadratic to the patch number, and the latter is linear when *M* is fixed (set to 7 by default). W-MSA and SW-MSA denote window-based multi-head self-attention using regular and shifted window partitioning configurations, respectively. Global self-attention computation is generally unaffordable for a large *hw*, while the window-based self-attention is scalable. *C* represents the channel of the input feature map.

For the global MSA module, it is assumed that the size of the input feature map is *H* × *W*, where *H* and *W* are the height and width of the feature map, respectively. The computational complexity of the global MSA module mainly depends on the self-attention calculation between all locations on the feature map. Therefore, the complexity can be expressed as

(3) $$O\left(N^{2}d\right)$$

where *N* = *H* × *W* is the number of positions in the feature map, and d is the dimension of each head. For the window-based MSA module, in window-based MSA, the input feature map is divided into non-overlapping windows of size *M* × *M*, where *M* is the size of the window. A self-attention calculation is performed inside each window, so the computational complexity of each window is

(4) $$O\left(M^{2}d\right)$$

Assuming there are *K* such windows, the computational complexity of the entire feature map is

(5) $$O\left(KM^{2}d\right)$$

With the shifted window partitioning approach, consecutive Swin-Transformer blocks are computed as

(6) $${\hat{z}}^{l}=W\text{-}\mathrm{MSA}\left(\mathrm{LN}\left(z^{l-1}\right)\right)+z^{l-1}$$

(7) $$z^{l}=\mathrm{MLP}\left(\mathrm{LN}\left({\hat{z}}^{l}\right)\right)+{\hat{z}}^{l}$$

(8) $${\hat{z}}^{l+1}=\mathrm{SW}\text{-}\mathrm{MSA}\left(\mathrm{LN}\left(z^{l}\right)\right)+z^{l}$$

(9) $$z^{l+1}=\mathrm{MLP}\left(\mathrm{LN}\left({\hat{z}}^{l+1}\right)\right)+{\hat{z}}^{l+1}$$

where ${\hat{z}}^{l}$ and *z^l^* denote the output features of the (S)W-MSA module and the MLP module for block *l*, respectively.

### 3.2. Model Structure

**Figure 1:**
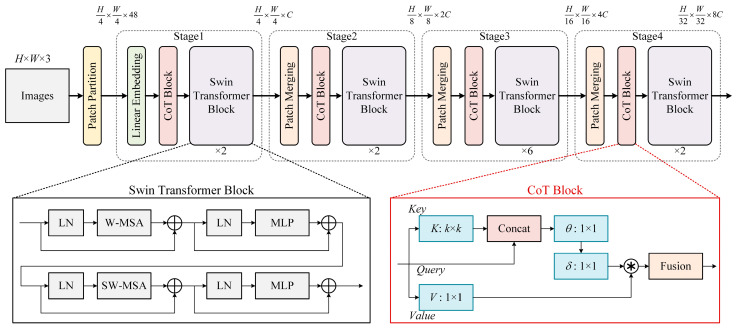
Overall structure diagram of the proposed method. ⊕ represents matrix sum and ⊛ represents matrix dot product.

The network proposed in this paper builds upon Swin-Transformer enhancements and introduces CoT attention to augment the feature extraction capabilities. The overall architecture is shown in Figure 1, characterized by a layered design with a total of four stages. At each stage, the resolution of the input feature map is systematically reduced, progressively expanding the receptive field layer by layer, similar to CNNs. Additionally, CoT-based blocks are integrated into each stage to enhance the local feature extraction capabilities.

The process begins with a *H* × *W* × 3 image (where *H* denotes height and *W* denotes width), which undergoes the patch partition operation to divide the image into tiles. These tiles are then embedded into an embedding layer to segment and encode the input image. Subsequently, in each stage, features are extracted through patch merging, CoT-based blocks, and Swin-Transformer-based blocks. The patch merging module is responsible for reducing the resolution of the feature map. The CoT-based block extracts local features by re-decomposing the feature map channels and fusing features while preserving the scale of the feature map. The Swin-Transformer block, composed of LayerNorm, MLP, window attention, and shifted window attention, plays a pivotal role in extracting global features from feature maps and fostering feature interactions between them.

While the traditional self-attention mechanism efficiently enables interactions among different spatial locations in the input, it has a limitation. In this mechanism, all pairwise query-key relationships are learned independently, without thoroughly exploring their rich contextual information. This restriction significantly hampers the capacity of self-attention mechanisms for visual representation learning across 2D feature maps. To address this challenge, we introduce the CoT attention module, which is designed to effectively mine contextual information.

The input feature map, denoted as *X* ∈ *R^H^*^×*W*×*C*^, has keys, queries, and values defined as *K* = *X*, *Q* = *X*, and *V* = *XW_v_*, respectively. In the CoT block, a *k* × *k* group convolution is initially applied spatially to all neighboring keys within a *k* × *k* grid, contextualizing each key representation. The *k* × *k* group convolution is a convolution layer in which the kernel is *k*. We set *k* to 3 for trade-off detection capabilities versus model complexity. The resultant learned context key *K*_1_ ∈ *R^H^*^×*W*×*C*^ inherently captures static contextual information among local neighbor keys. *K*_1_ is considered the static context representation of the input *X*. Subsequently, conditional on the concatenation of the context key *K*_1_ and the query *Q*, the attention matrix is derived through two consecutive 1 × 1 convolutions (i.e., *W_θ_* with ReLU activation function and *W_δ_* without activation function):

(10) $$A=\left[K_{1},Q\right]W_{\theta }W_{\delta }$$

Next, according to the contextual attention matrix *A*, we calculate the participation feature map *K*_2_ by aggregating all values *V*:

(11) $$K_{2}=V⊙A$$

Among them, *K*_2_ is the dynamic context representation of the input, which captures the dynamic feature interaction between inputs, and the ⨀ denotes the local matrix multiplication operation that measures the pairwise relations between each query and the corresponding keys within the local grid in space.

Therefore, the final output of CoT can measure the fusion result of static context *K*_1_ and dynamic context *K*_2_.

### 3.3. Ancient Chinese Data Enhancement Strategy

**Figure 2:**
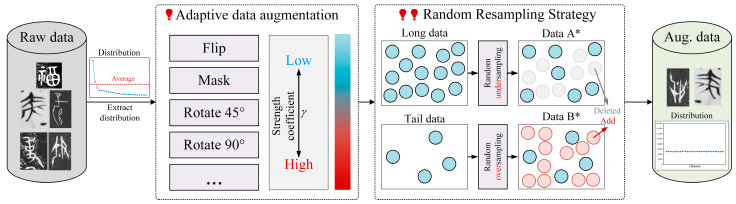
Ancient Chinese character data enhancement strategy. The proposed strategy is divided into two steps: (1) in the first step we will perform adaptive data enhancement on the original data, and (2) in the second step we will randomly resample the data (Aug. data stands for Augment data). * represents augmented data.

As shown in Figure 2, there is a long-tail phenomenon in the distribution of ancient Chinese data. This paper proposes an enhancement strategy. Initially, we calculated the distribution of data classes using the original ancient Chinese character data. The original data exhibit a significant long-tail distribution issue, whereas most of the dataset is dominated by a few head classes, leaving a smaller portion for the tail data. We established a strength coefficient, denoted as *γ*, derived from the distribution of the original data. This coefficient serves as a crucial factor in regulating the magnitude of data enhancement operations, such as flipping, random masking, random rotation, and others. A higher value of *γ* corresponds to a more pronounced and robust data enhancement effect. We adopted an enhancement strategy to reduce the reinforcement rate of the head data *γ*, effectively increasing the reinforcement ratio of tail data. This approach aims to initially address the long-tail distribution within the dataset. It is noteworthy that applying data augmentation to the entire dataset, as opposed to solely the tail data, is intentional. This choice helps avoid potential confusion in the feature distribution of the dataset that may arise if data augmentation is selectively applied to only a portion of the dataset. The reinforcement ratio *γ* can be calculated as:

(12) $$\gamma =1-\frac{n_{i}}{\sum_{j=0}^{N}n_{j}},$$

where *n_i_* represents the number of character instances of the *i*-th class, and *N* represents the number of classes in the ancient Chinese character dataset. It can be known from the formula that the larger *n_i_* is, the smaller *γ* is.

We developed a random resampling strategy to introduce variability to the dataset. Initially, we calculated the average number of classes in the original dataset. Subsequently, leveraging this average, we randomly oversampled the tail data and undersampled the head data. This can result in a balanced number of augmented data samples for each class. As shown in Figure 2, it demonstrates the effectiveness of the proposed ancient Chinese data enhancement strategy in addressing the long-tail distribution issue inherent in the ancient Chinese character dataset.

## 4. Experiments, Analysis, and Discussion

### 4.1. Datasets

#### 4.1.1. Dataset of Sample Acquisition

**Figure 3:**
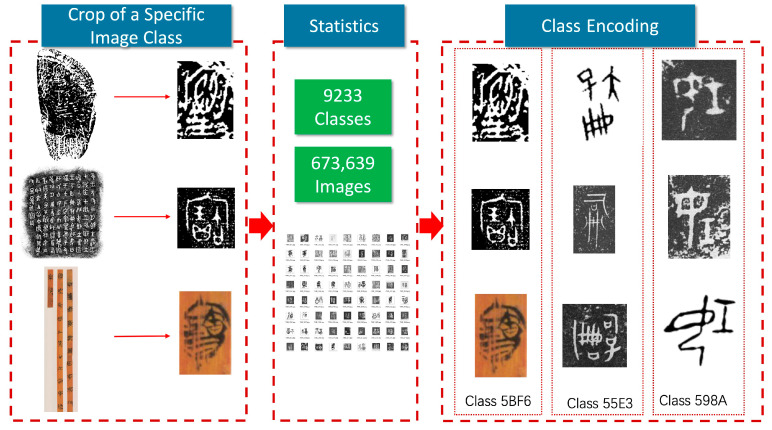
Process of collecting ancient Chinese characters from unearthed documents.

We collected the ancient Chinese character imagery from the Shang to Qin dynasties in a relatively comprehensive manner, including oracle bone inscriptions, bronze inscriptions, bamboo and silk scripts, coinage scripts, and stone engravings. As shown in Table 3, the relevant dictionaries or character compilations listed are representative research achievements in the field of literature studies. As shown in Figure 3, we obtained the original image data of the character materials by scanning or taking photos. Most of these character materials are rubbings of inscriptions, while a few bamboo and silk scripts are colored photographs. In each cropped sample, there is only one ancient Chinese character. We grouped identical characters together as a class and assigned a unique code to each class. Subsequently, we obtained the original dataset.

**Figure 4:**
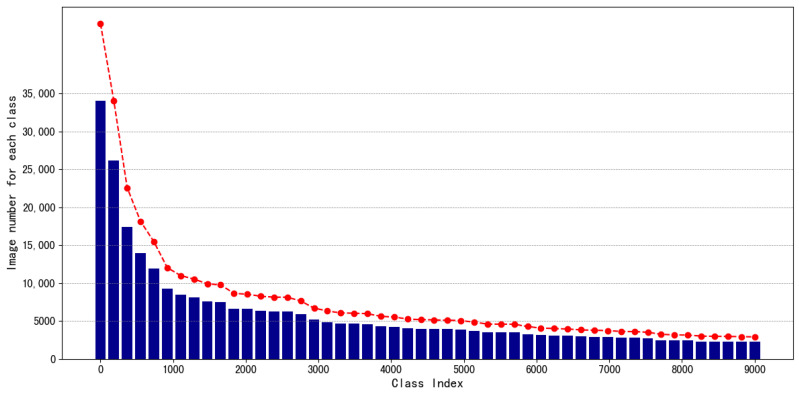
Number of images in different classes of the ancient Chinese character dataset showing a long-tail distribution (classes shown in the figure are post-sampling categories, and the red lines indicate the trend of changes in the number of instances for each class.).

The original dataset contains 673,639 images spanning 9233 classes. On average, there are 51 images per class in the training set. Due to the power-law distribution characteristic of the frequency of character usage, the distribution of the number of images also exhibits a long-tail effect: the class with the most images has 34,000, while the one with the fewest has only 2 images. As shown in Figure 4, this dataset is unbalanced. The dataset is named the “Ancient Chinese Character Dataset”. After the collecting of raw data, we enhanced the dataset completeness and diversity through data preprocessing and data enhancement.

#### 4.1.2. Data Preprocessing

**Figure 5:**
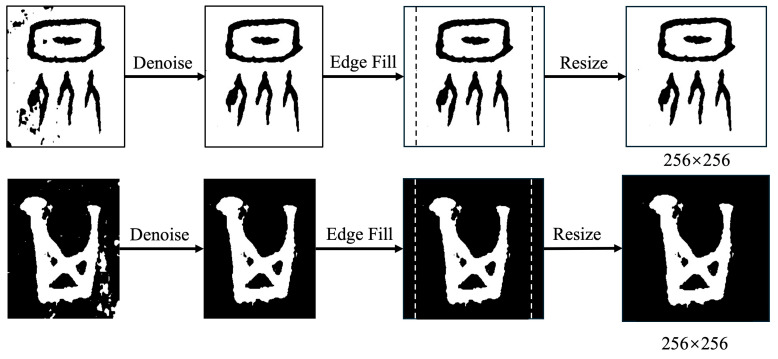
The preparation process of ancient Chinese characters “众” (crowd) and “其” (its/his/her/their).

To extract the calligraphic features of ancient Chinese characters more accurately, we preprocessed the raw samples. The preprocessing procedure is illustrated in Figure 5.

- Denoising: In the raw images of ancient Chinese characters, there is a significant amount of “salt and pepper noise”. We first employed a median filter to remove noise. This method sorts all pixel values around each pixel in the image and assigns the middle value (i.e., the median) to the central pixel, effectively eliminating noise. This approach is particularly suitable for removing salt and pepper noise as it preserves image edges effectively without causing blurring.
- Size standardization: We standardized the image size to 256 × 256 pixels while preserving the original aspect ratio. For non-square images, we first padded the edges with white or black pixels and then adjusted them to the desired size.

#### 4.1.3. Data Enhancement

We implemented the data enhancement strategy proposed in Section 3.3 to expand the original dataset, addressing the long-tail distribution issue. It should be noted that, to balance computational efficiency and data balance, we did not intentionally equalize the number of samples for all 9233 classes but rather focused on enhancing the sample size of the tail-end data. After completing the enhancement operations, the class with the fewest samples in the training set contained 50 images. The total number of samples in the dataset increased from 673,639 to 979,907. The ratio between the training and test sets was close to 4:1. In the training set, the imbalance ratio n_max_:n_min_ was 477:1, where n_max_ and n_min_ represent the classes with the most and fewest samples, respectively. In contrast, before the enhancement operation, the imbalance ratio in the training set reached 23,828:1. Therefore, the problem of long-tail distribution has been alleviated to some extent.

After the above steps, we have completed the construction of the “Ancient Chinese Character Dataset”.

### 4.2. Experimental Setup

The experimental environment configuration of the method proposed in this article is based on Ubuntu16.04.1 (Linux 4.15.0-142-generic). The server was equipped with an Intel CPU (Intel(R) Xeon(R) Gold 6242R CPU @ 3.10 GHz) and eight NVIDIA GPU (GeForce RTX 3090) with 24 GB of video memory and 256 GB of memory. The server was purchased from Huamei Technology (Suzhou) Co., Ltd., Suzhou, China. The training batch size was set to 32 and the epoch to 20. The optimizer used was AdamW, with an initial learning rate of 0.001. The typical time it takes per epoch is about 1–2 h, and the inference time per letter is 53.6 ms.

### 4.3. Comparison Experiments

We conducted a performance evaluation experiment using the proposed method on the collected ancient Chinese text dataset and compared it with the latest target classification model. The comparative experimental results can be found in Table 4. It can be seen that the proposed method achieves 87.2469% and 95.8088% in top-one accuracy and top-five accuracy, respectively, under the image size of 256 × 256, reaching the state-of-the-art level. This proves that the proposed structure has significantly enhanced the potential for text feature extraction. Compared with the baseline, the method in this article improves the top-one accuracy by 1.5074% and the top-five accuracy by 0.9416%. Experimental results show that the ancient Chinese character classification model achieves superior results in classifying ancient characters. We also compared the performance capabilities of each model under different image sizes. It can be found that the method proposed has a top-one accuracy of 86.6273% under an image size of 224 × 224 and a top-one accuracy under 256 × 256. The accuracy is 87.2569%, an increase of 0.6196%. The results indicate that the model exhibits varying performance across different image sizes.

### 4.4. Ablation Experiments

In this study, we conducted ablation experiments to systematically assess the impact of our proposed methods on the recognition performance, as detailed in Table 5. We adopted the Swin-Transformer as a baseline model to evaluate the effectiveness of the data augmentation strategy. The CoT block was introduced to enhance the model’s classification capabilities.

The results presented in Table 5 reveal that employing the data augmentation strategy alone on the dataset yielded a significant improvement in the model’s top-one accuracy, which reached 86.2664%, marking an increase of 0.5269% compared to the baseline. Furthermore, the combination of the data augmentation strategy and the CoT block led to a substantial enhancement in the model’s classification performance. The top-one accuracy experienced a notable increase of 1.5074%.

These findings underscore the effectiveness of the data augmentation strategy and the CoT block in boosting the model’s classification capabilities, emphasizing their potential as valuable components in the development of more accurate and robust classification models.

### 4.5. Visual Analysis

**Figure 6:**
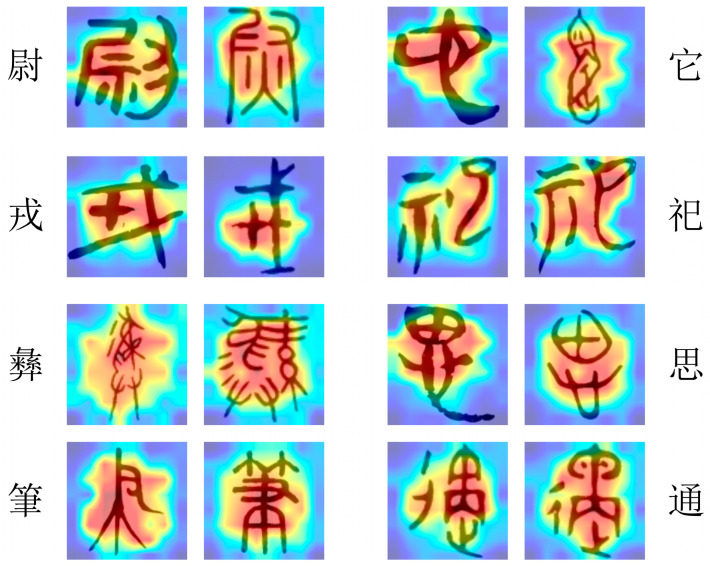
Heat map classification results of the method proposed in this article on different ancient characters. From top to bottom on the left side, we have “尉” (military officer), “戎” (ancient weaponry), “彝” (ceremonial vessel), and “笔” (pen or writing instrument). On the right side, from top to bottom, we have “它” (it), “祀” (sacrifice), “思” (think), and “通” (communicate).

We utilized a heat map to visualize the model’s classification performance. We mapped the attention layer of the last Swin-Transformer block of the network by using the GradCAM method [58]. The red part represents the place where the attention is strongest, and the blue part represents the place where the attention is weakest. The heat map presented in Figure 6 is an intriguing aspect of this study. The visualization technique offers a profound insight into the recognition behavior—specifically, how it attends to different ancient Chinese characters.

The heat map effectively highlights the regions of the characters that the model focuses on the most. In the context of Chinese characters, this is particularly significant because Chinese script is composed of complex shapes and strokes, each with distinctive features that contribute to its identity. The fact that the model can prioritize the shape of the characters, regardless of the background, is a testament to its sophistication and accuracy. This ability to discern and prioritize the relevant features of Chinese characters is crucial for accurate classification. The attention mechanism ensures that it does not get distracted by irrelevant details or background noise, but instead focuses squarely on the characteristics that define each character. This not only enhances the model’s classification accuracy but also provides a transparent understanding of its decision-making process.

**Figure 7:**
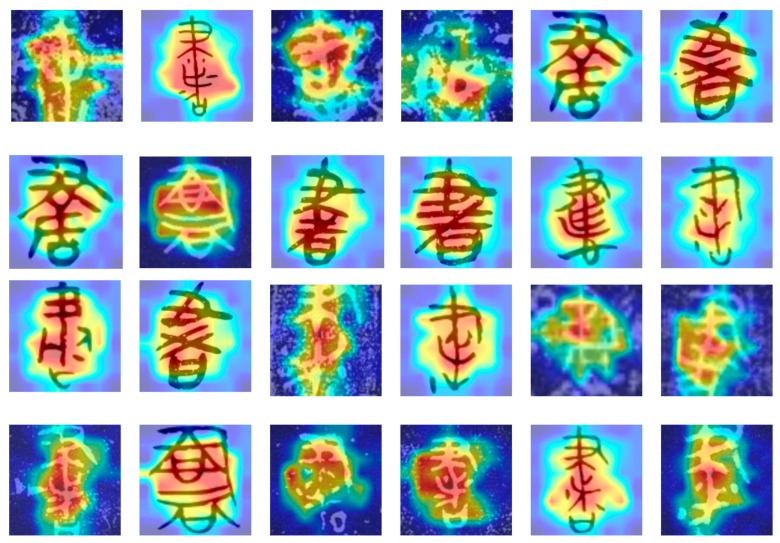
The attention visualization results of the ancient character classification model proposed in this article on the word “书” (book) of various ages.

**Figure 8:**
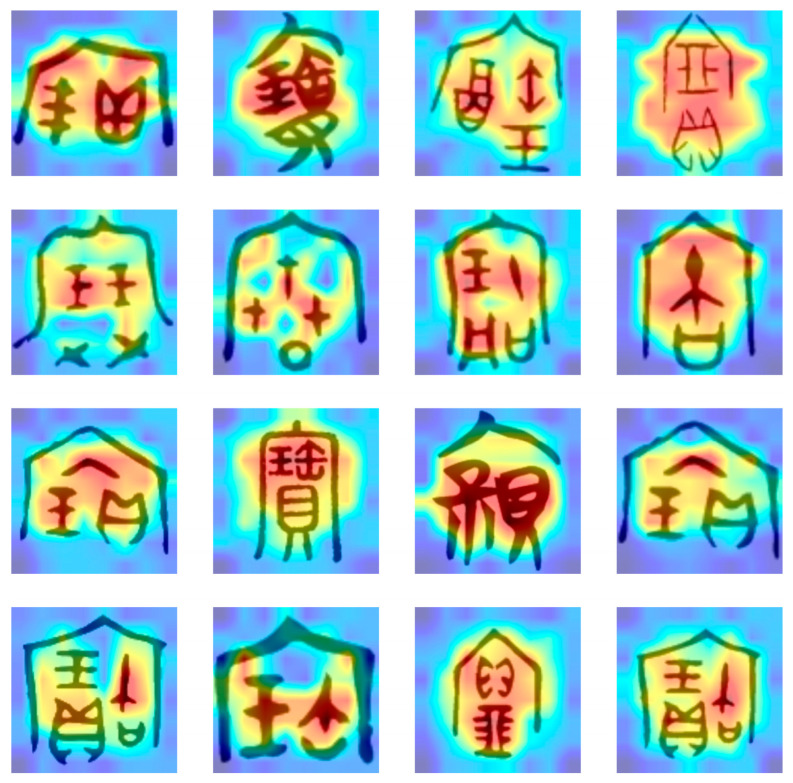
The attention visualization results of the ancient character classification model proposed in this article on the word “宝” (treasure) of various ages.

We further analyzed the visual attention mechanism of Chinese characters with significant font changes in the dataset, aiming to explore the ability of the proposed model to recognize different font style features. Specifically, we selected the characters “书” (book) and “宝” (treasure) as case studies for this investigation. As shown in Figure 7 and Figure 8, we can find the focal points of the model when processing Chinese characters with diverse font styles from various historical periods and backgrounds.

The attention visualization results demonstrate that the proposed model can effectively identify key feature regions of Chinese characters, maintaining robust recognition performance even in scenarios with diverse and complex font styles. The areas highlighted in red represent the regions to which the model pays the most attention. These regions often contain the most distinctive features crucial for character recognition. In contrast, the blue areas indicate regions that the model deems less relevant. This visualization technique offers an intuitive way to comprehend the model’s operations and validates the effectiveness of the proposed method in handling the task of recognizing Chinese characters with diverse font variations.

### 4.6. Discussion

The remarkable recognition performance may be attributed to our proposed model effectively learning the textual characteristics of ancient Chinese characters from a large-scale dataset. In other words, the model learned from a vast number of images to understand the distribution patterns of ancient Chinese character forms, achieving successful recognition outcomes. The model can effectively distinguish between single-component characters (composed of a single component) and compound characters (i.e., composed of multiple components). It can also capture the features of each component of ancient Chinese characters and understand the meaning conveyed by their combinations.

**Figure 9:**
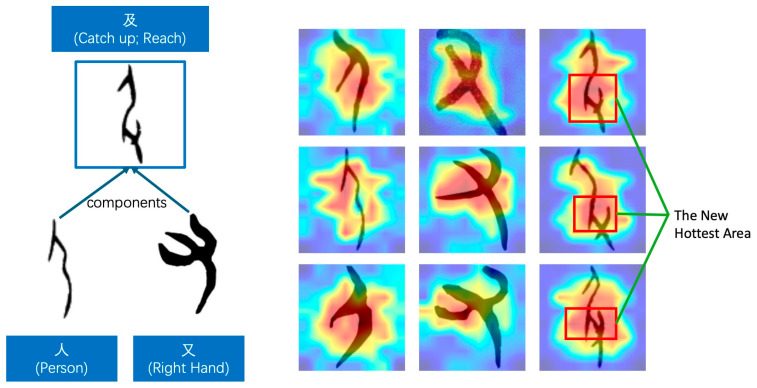
The composition and the hottest area of the heatmap for the ancient Chinese character “及”.

Taking the character “及” (catch up, reach) as an example, as shown in Figure 9, it consists of two components, “人” (person) and “又” (right hand). The meaning of these characters is represented by the combination of two components: a hand grabbing the leg of a person, indicating “catch up” or “arrive”. The component “人” resembles a person slightly bowing with their arms naturally hanging down. The “又” component represents the “right hand”, appearing like an open hand (only with three fingers for ease of writing). When each component is individually recognized, the model’s highest attention area (the reddest area in the heatmap) is approximately at the “separated part between the downward arm and the torso” for “人” and at the three open fingers for “又”. These are the most distinguishing features of these two components. However, when the combination of “人” and “又” represents the character “及”, we found that the central region of the decision is not a simple concatenation of the highest attention areas of the two components. Instead, it is at the part where “the hand grabs the person’s leg”. This indicates that the model can distinguish between single-component and compound characters and, to some extent, accurately learn the meaning of the character “及”.

**Figure 10:**
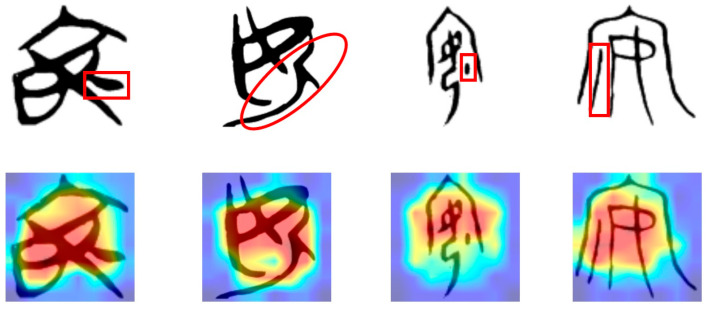
The main component of the ancient Chinese character “安” which has been previously overlooked—the red box or circle indicator—is located at the focal point of the model’s attention.

The proposed model even pays attention to the original meaning of Chinese characters when recognizing them. Taking the Chinese character “安” (safe) as an example, the existing research experience suggests that the character “安” expresses “a woman sitting indoors” (i.e., the upper part of the character “宀” represents a house, and the character “女” represents a woman). Thus, the character “安” is then extended to the common meaning of “safety, peace”. However, recent research has found that this view is not entirely accurate. The original meaning of “安” should be “a woman sitting with her feet tucked underneath her”. In early versions of the character “安”, where a dot or a horizontal stroke below the “女” component indicated the “feet”, this element was often considered a meaningless decorative stroke in previous studies. The “宀” component was added later to enhance the expressiveness of the character. From the heatmap generated by the model, as shown in Figure 10, it can be observed that the model focuses on the stroke below the “女” component in the character “安” during classification. However, the component “宀”, representing a house, did not receive the highest attention. This aligns with the views of ancient Chinese scholars and confirms that the stroke is not merely a meaningless decoration but a distinctive feature with semantic significance in character formation.

## 5. Conclusions

Identifying ancient Chinese scripts is of great significance for cultural heritage and historical research. To address the challenge of recognizing ancient Chinese characters, we constructed a large-scale image dataset of common ancient Chinese characters comprising 9233 distinct characters derived from images obtained from archaeological excavations. The dataset encompasses nearly all common ancient scripts. We proposed an improved Swin-Transformer model which consists of four stages with Linear Embedding and Swin-Transformer blocks, each comprising Linear Embedding, CoT block, and Swin-Transformer blocks. The CoT Block was utilized to enhance the feature learning ability to extract local features. Extensive experiments were conducted on the ancient Chinese characters dataset. The experimental results, with a top-one accuracy of 87.25% and a top-five accuracy of 95.81%, demonstrate that our proposed method achieves outstanding performance on the dataset, outperforming other algorithms and reaching state-of-the-art accuracy. The next step of this work is to explore how to integrate the structural characteristics and radical features into the design of recognition models.

## Figures and Tables

**Table 1:** Ancient Chinese characters from different periods, illustrated with examples of “马 (horse)”, “牛 (ox)”, “明 (bright)”, and “走 (run)”.

Representative Script Types	Period	马	牛	明	走
Oracle bone inscriptions; bronze inscriptions	Shang Dynasty (about 1700 B.C.E.—1100 B.C.E)	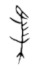	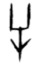	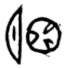	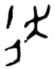
Bronze inscriptions	West Zhou Dynasty (about 1100 B.C.E.—770 B.C.E)	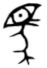	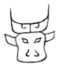	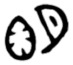	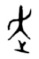
Bronze inscriptions; stone inscriptions; pottery inscriptions; bamboo slip, wooden tablet, and silk manuscript texts	Spring and Autumn Period (about 770 B.C.E.—476 B.C.E)	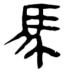	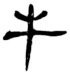	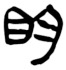	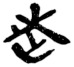
Bronze inscriptions; seal inscriptions; coin inscriptions; bamboo slip and silk manuscript texts	Warring States Period (about 476 B.C.E.—221 B.C.E)	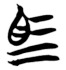	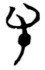	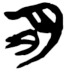	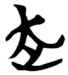

**Table 2:** Writing styles of the characters “者 (someone who)” and “市 (market)” in the states of Qin, Chu, Qi, Yan, and Jin.

Modern Chinese Characters	Qin	Chu	Qi	Yan	Jin
者 zhě	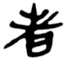	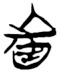	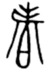	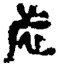	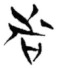
市 shì	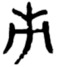	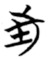	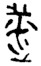	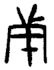	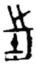

**Table 3:** Source of ancient character materials and examples of various ancient characters, such as “王 (King)”, “君 (Ruler)”, “云 (Cloud)”, and “疑 (Doubt)”.

Source Materials of Ancient Chinese Characters	Literature	Example			
Oracle bone inscriptions	[35,36,37,38]	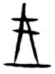	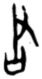	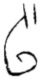	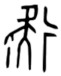
Bronze inscriptions	[38,39,40,41,42,43,44,45]	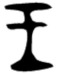	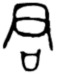	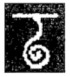	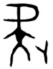
Seal inscriptions	[38,42,44,45]	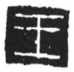	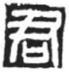	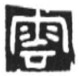	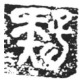
Coin inscriptions	[38,41,42,43,44,45]	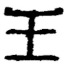	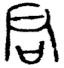	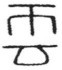	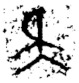
Pottery inscriptions	[38,41,42,43,44,45]	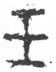	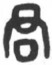	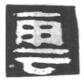	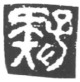
Bamboo slip and silk manuscript texts	[38,45,46,47,48]	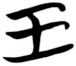	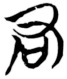	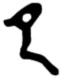	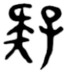

**Table 4:** Comparison with other methods. **Bold** text indicates the best accuracy, and underlined text indicates second best accuracy.

Models	Backbone	Image Size	Top-1 Accuracy	Top-5 Accuracy
ResNet101 [23]	ResNet101	224 × 224	84.1283	94.6614
ResNet101 [23]	ResNet101	256 × 256	83.0917	94.1338
SwinT-v2 [34]	SwinTv2-small	256 × 256	85.7395	94.8672
Conformer [49]	Conformer-base	256 × 256	82.1387	93.3345
Convnext [50]	Convnext-base	256 × 256	81.2875	92.8199
DenseNet [24]	DenseNet121	256 × 256	76.6406	90.3361
EfficentNet [26]	EfficientNet-b3	224 × 224	77.4249	91.1524
Poolformer [51]	Poolformer-s24	224 × 224	78.4632	90.9764
RepVGG [52]	Repvgg-A0	224 × 224	80.1162	92.2734
Res2Net [53]	Res2Net-101	224 × 224	77.3113	90.8053
ResNeSt [54]	ResNeSt-101	256 × 256	83.1570	94.0690
ResNeXt [55]	ResNeXt-101	224 × 224	74.0102	88.8082
SeResNet [56]	SeResNet-101	224 × 224	71.7029	86.5144
ShuffleNet-v2 [57]	ShuffleNet-v2	224 × 224	79.4450	91.7922
Ours	Ours-backbone	224 × 224	86.6273	95.1640
Ours	Ours-backbone	256 × 256	**87.2469**	**95.8088**

**Table 5:** Ablation experiment.

+Enhancement Strategy	+CoT Block	Top-1 Accuracy	Top-5 Accuracy
		85.7395	94.8672
√		86.2664	95.2735
	√	86.6730	95.7355
√	√	87.2469	95.8088

## Data Availability

The data generated during this study are currently private due to the fact that our team is about to have a new breakthrough in this study. The data will be made public soon.
